# A235 DETERMINANTS AND OUTCOMES OF EARLY CHOLECYSTECTOMY FOR GALLSTONE PANCREATITIS IN BRITISH COLUMBIA.

**DOI:** 10.1093/jcag/gwae059.235

**Published:** 2025-02-10

**Authors:** A A Arif, S Sasson, N Aboalfaraj, J Telford

**Affiliations:** Internal Medicine, The University of British Columbia Faculty of Medicine, Vancouver, BC, Canada; Internal Medicine, The University of British Columbia Faculty of Medicine, Vancouver, BC, Canada; The University of British Columbia, Faculty of Medicine, Division of Gastroenterology, Vancouver, BC, Canada; The University of British Columbia, Faculty of Medicine, Division of Gastroenterology, Vancouver, BC, Canada

## Abstract

**Background:**

Gallstone pancreatitis is the most common cause of pancreatitis, with early cholecystectomy reducing recurrence and improving outcomes compared to delayed surgery or ERCP alone. However, the use of early cholecystectomy in British Columbia (BC) and its long-term effects are not well understood.

**Aims:**

This study will evaluate the prevalence and outcomes of early cholecystectomy in BC, potentially contributing to improved management guidelines.

**Methods:**

A retrospective multicenter analysis of patients with gallstone pancreatitis admitted from 2018 to 2023 to four hospitals within BC. Patients with prior cholecystectomy or pancreatic cancer were excluded from the analysis. Patients with moderate or severe pancreatitis as determined by the revised Atlanta criteria were excluded. Patients who received a cholecystectomy in under 4 weeks were labelled as early, whereas over 4 weeks or not at all were labelled as late. Baseline characteristics, details of cholecystectomy and outcomes were recorded.

**Results:**

A total of 193 patients with a mean age of 58.5 years (SD 18.4) and 60.1% female were included. 84 patients underwent early cholecystectomy (43.5%) including during the index admission (36.3%). Patients who received early cholecystectomy were younger (52.6 years vs 62.9 years, P<0.001) with fewer comorbidities (Charlson comorbidity index 1.5 vs 2.8, P<0.001). Patient gender, body mass index or hospital type were not associated with early cholecystectomy. Early cholecystectomy patients were more likely admitted by a surgeon (OR 11.7, 95% CI 4.6 to 29.6, P<0.001), offered cholecystectomy at index admission (OR 59.8, 95% CI 24.5 to 146.0, P<0.001), and receive cholecystectomy at index admission (OR 497, 95% CI 64.1 to 3848, P<0.001). There was no difference in the symptom onset to admission time interval or length of stay. ERCP was equally likely in both groups (OR 0.62, 95% CI 0.34 to 1.1, P=0.12). Patients who received early cholecystectomy were less likely to have a recurrent biliary event (HR 0.26, 95% CI 0.15 to 0.45, P<0.001). Pancreatitis was the most common (81.1%) recurrent biliary event. Reasons for not undergoing early cholecystectomy included: referral to outpatient cholecystectomy as the most common (29.7%), age and/or comorbidities (25.0%) and patient declining surgery (21.9%).

**Conclusions:**

Early cholecystectomy reduces recurrent rates of biliary events. Admission under surgery and offering surgery at the index admission were much more likely in the early group. Early surgical involvement may be an important factor in achieving higher rates of index admission cholecystectomy.

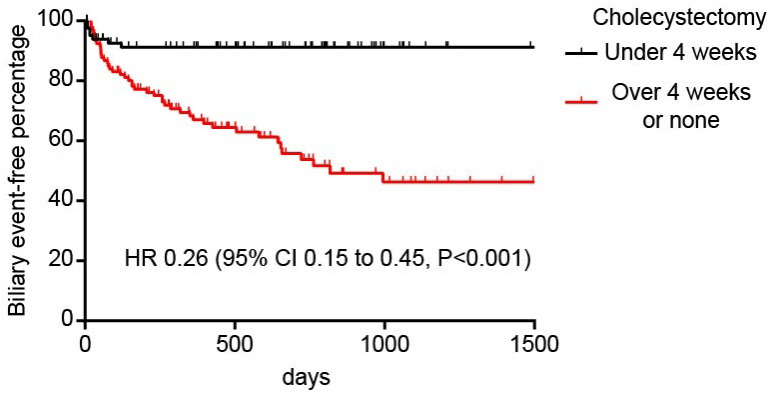

**Funding Agencies:**

None

